# Situs Inversus Totalis with Mirror-Image Dextrocardia and Acute Type A Aortic Dissection Treated by Ascending Aorta Replacement Plus Total Arch Replacement and Frozen Elephant Trunk Using an Inverted Four-Branched Graft: A Case Report

**DOI:** 10.3390/jcm15218500

**Published:** 2026-11-01

**Authors:** Lile He, Zhiwei Zhang, Tao Tang

**Affiliations:** Department of Cardiovascular Surgery, The Second Xiangya Hospital, Central South University, Changsha 410011, China

**Keywords:** situs inversus, dextrocardia, aortic dissection, total arch replacement, frozen elephant trunk

## Abstract

Situs inversus totalis (SIT) is a rare congenital condition characterized by a complete mirror-image reversal of the thoracic and abdominal viscera, usually accompanied by dextrocardia. Acute type A aortic dissection (AAD) is a highly lethal cardiovascular emergency, and its coexistence with SIT is exceedingly rare. To the best of our knowledge, apart from a recently reported pregnant patient, no case of AAD in a non-pregnant adult with SIT has been treated with ascending aorta replacement combined with total arch replacement (TAR) and frozen elephant trunk (FET) implantation. In this report, we present such a case and share our surgical experience, particularly the inverted four-branched graft technique used to resolve the mismatch between the commercially available graft and the mirrored anatomy. The postoperative course was complicated by right renal malperfusion and stage 2 acute kidney injury. Overall renal function recovered with conservative management, although the right kidney subsequently became atrophic; otherwise, recovery was satisfactory. Computed tomographic angiography (CTA) at 10 months showed the graft and all reconstructed branches patent and the FET in position without endoleak, and the patient remained in good condition at the telephone follow-up approximately 3 years after surgery.

## 1. Introduction

Mirror-image dextrocardia is characterized by the heart being situated on the right side of the chest, with its major structures mirrored from their normal positions [1]. This condition frequently coexists with situs inversus, in which the other thoracic and abdominal organs are also transposed, maintaining relative symmetry and potentially mitigating associated complications [2,3]; when the inversion involves all of the thoracic and abdominal viscera, the condition is defined as situs inversus totalis (SIT) [2]. The incidence of mirror-image dextrocardia is approximately 1 in 12,000 [1], and its clinical presentation can vary considerably, ranging from complex congenital heart disease to life-threatening cardiovascular conditions requiring medical intervention [3].

Acute type A aortic dissection (AAD) is a highly fatal cardiovascular disease [4]. It occurs acutely, predominantly in individuals around 50 years of age, with an annual incidence ranging from approximately 3.5 to 15 per 100,000, which is increasing over time [5]. Without treatment, the mortality of AAD may reach 90%, and even with prompt surgical treatment, the in-hospital 30-day mortality rate remains as high as 15–25% [4,5]. Although the application of TAR combined with FET in AAD still remains controversial, it has evolved into an accepted treatment option for AAD patients [6,7]. Extended arch replacement may promote favorable distal aortic remodeling; however, whether it confers a long-term survival benefit over limited arch repair remains to be determined [8,9]. Apart from a recently reported pregnant patient with SIT [10], to the best of our knowledge, no non-pregnant adult with mirror-image dextrocardia and AAD has previously undergone this procedure. We herein report a case of AAD in a patient with SIT and mirror-image dextrocardia successfully treated with ascending aorta replacement plus TAR with FET, focusing on the anatomical and technical challenges posed by the mirrored anatomy and on the inverted four-branched graft technique used to resolve them.

## 2. Case Presentation

This case report was prepared in accordance with the CARE (CAse REport) guidelines.

### 2.1. Clinical Presentation and Investigations

A 50-year-old male presented to the emergency department on the evening of 23 October 2023 with sudden, persistent tearing pain in the chest and back lasting 6 h.

On admission, his vital signs and physical measurements were as follows: body temperature 36.3 °C, pulse rate 68 beats per minute, and respiratory rate 20 breaths per minute, with a height of 168 cm and a weight of 74.0 kg. Blood pressure was 131/56 mmHg in the left upper limb, 155/62 mmHg in the right upper limb, 91/43 mmHg in the left lower limb, and 94/44 mmHg in the right lower limb. On cardiovascular examination, the apical impulse was detected at the fifth intercostal space, approximately 0.5 cm medial to the right midclavicular line. No abnormal pulsations or pericardial friction rub were identified on auscultation or palpation, and percussion revealed heart borders within normal limits. Heart sounds were clearly audible within the right thoracic cavity, with no audible murmurs over the cardiac valve areas.

The patient had a 10-year history of hypertension and had been on long-term antihypertensive medication, with systolic blood pressure typically ranging from 140 to 160 mmHg. No other significant medical history was reported.

**Figure 1:**
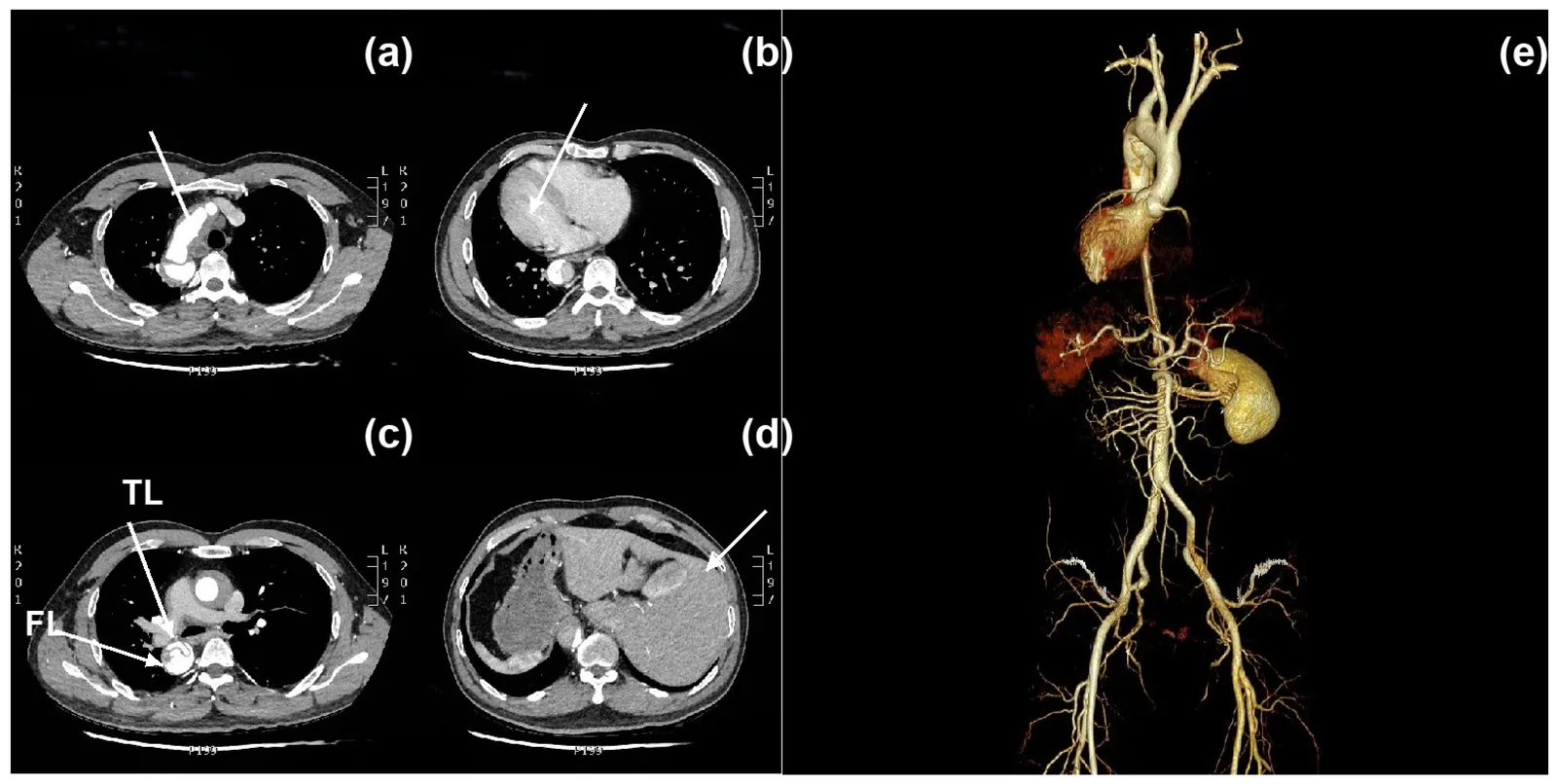
Preoperative CTA findings. (**a**) Axial image showing the intimal flap in the ascending aorta (arrow). (**b**) Axial image showing mirror-image dextrocardia (arrow indicates the right ventricle). (**c**) Axial image showing the intimal flap in the descending aorta, with the true lumen (TL) and the false lumen (FL) (arrow). (**d**) Axial abdominal image demonstrating complete situs inversus, with the liver on the patient’s left side (arrow). (**e**) Three-dimensional volume-rendered reconstruction showing the mirror-image dextrocardia and the dissected aorta.

Given the sudden onset of persistent tearing pain in the chest and back, acute myocardial infarction and pulmonary embolism were considered important differential diagnoses. Echocardiography revealed a floating intimal flap in the ascending aorta, indicative of type A aortic dissection, as well as mirror-image dextrocardia with normal left ventricular systolic function. Computed tomographic angiography (CTA) confirmed aortic dissection involving the ascending aorta, aortic arch, and descending aorta, with the primary entry tear located in the descending aorta (Figure 1; Supplementary Materials, Video S1). In addition, CTA demonstrated complete visceral transposition, with the liver located in the left hypochondrium, the spleen and stomach on the right, and the inferior vena cava to the left of the midline, consistent with SIT (Figure 1). The diagnoses were therefore established as follows: (1) acute type A aortic dissection; (2) mirror-image dextrocardia; and (3) situs inversus totalis.

### 2.2. Surgical Technique

The patient underwent emergency ascending aorta replacement plus TAR with FET, as previously described by our team [11]. Intraoperative findings revealed visceral malposition, with the superior and inferior vena cava located on the left side of the midline and the cardiac apex directed towards the right lower side. Approximately 100 mL of hemorrhagic effusion was aspirated from the pericardium. The dissection involved the ascending aorta, aortic arch, and descending aorta, proximally affecting the non-coronary and right coronary sinuses of the aorta, and distally involving all three branches of the aortic arch and extending into the descending aorta. The aortic valve leaflets remained intact, without valvular regurgitation. The operation was performed on 24 October 2023. After induction of general anesthesia, central venous catheterization was performed via the left internal jugular vein; this route was selected to keep the right side of the neck and chest wall accessible in the setting of mirrored mediastinal anatomy, although the course of the thoracic duct-which may itself be variable or mirrored in SIT-could not be confirmed preoperatively. No intimal tear was identified in the ascending aorta or aortic arch, and the false lumen contained abundant thrombus; the primary entry tear was identified in the descending aorta on preoperative CTA.

**Figure 2:**
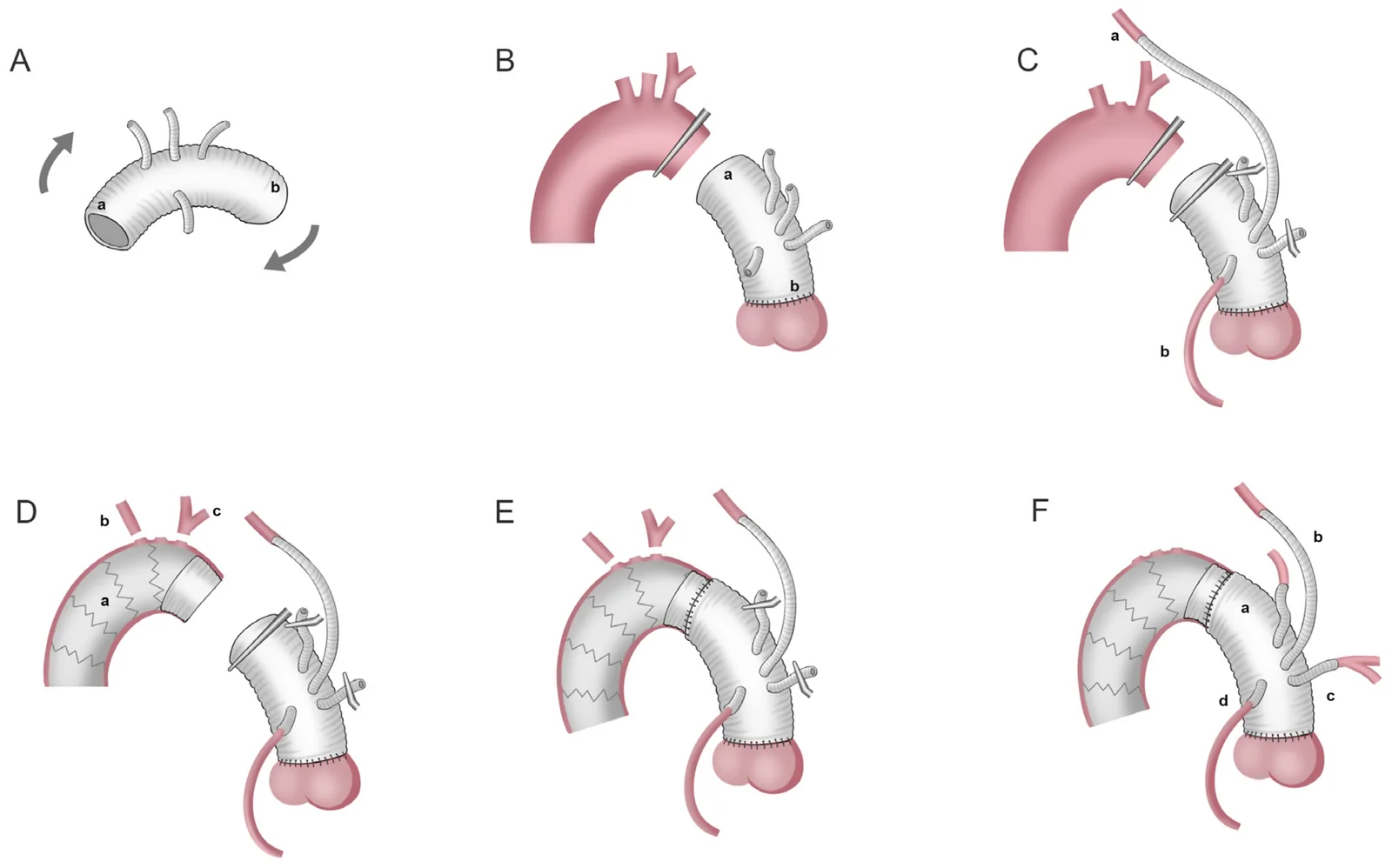
Schematic illustration of the operative procedure using the four-branched woven graft implanted in an inverted orientation. In panels (**A**,**B**), a denotes the proximal end of the graft and b its distal end; panels (**C**–**F**) define their own labels in the corresponding sentences. (**A**) The four-branched prosthetic graft before implantation, with its proximal end (a) and distal end (b) marked; it is subsequently implanted in an inverted orientation, so that its distal end (b) is anastomosed to the aortic root while its proximal end (a) is used for the distal anastomosis. The arrows indicate this change in orientation. (**B**) In the implanted orientation, the distal end of the graft (b) is anastomosed to the aortic root, while its proximal end (a) is oriented toward the aortic arch. (**C**) Selective perfusion during lower-body circulatory arrest: the second branch, already anastomosed to the right common carotid artery, delivers unilateral antegrade selective cerebral perfusion (a), while the fourth branch (b) is connected to the arterial perfusion line of the cardiopulmonary bypass circuit to maintain the blood supply of the heart and, through the second branch, of the right cerebral hemisphere; the open distal end of the graft and the unused branch stumps are occluded with clamps to prevent runoff. (**D**) The frozen elephant trunk (a)-a covered stent graft with a short woven polyester cuff at its proximal end (Cronus; MicroPort Endovascular, Shanghai, China)-is deployed into the descending aorta, and the innominate artery (c) and the right subclavian artery (b) are divided. (**E**) The proximal end of the graft is anastomosed end-to-end to the woven polyester cuff of the frozen elephant trunk (distal anastomosis). (**F**) Appearance after completion of the main operative steps: a, the reconstructed right subclavian artery; b, the right common carotid artery; c, the innominate artery; d, the fourth branch of the graft (arterial perfusion line).

**Figure 3:**
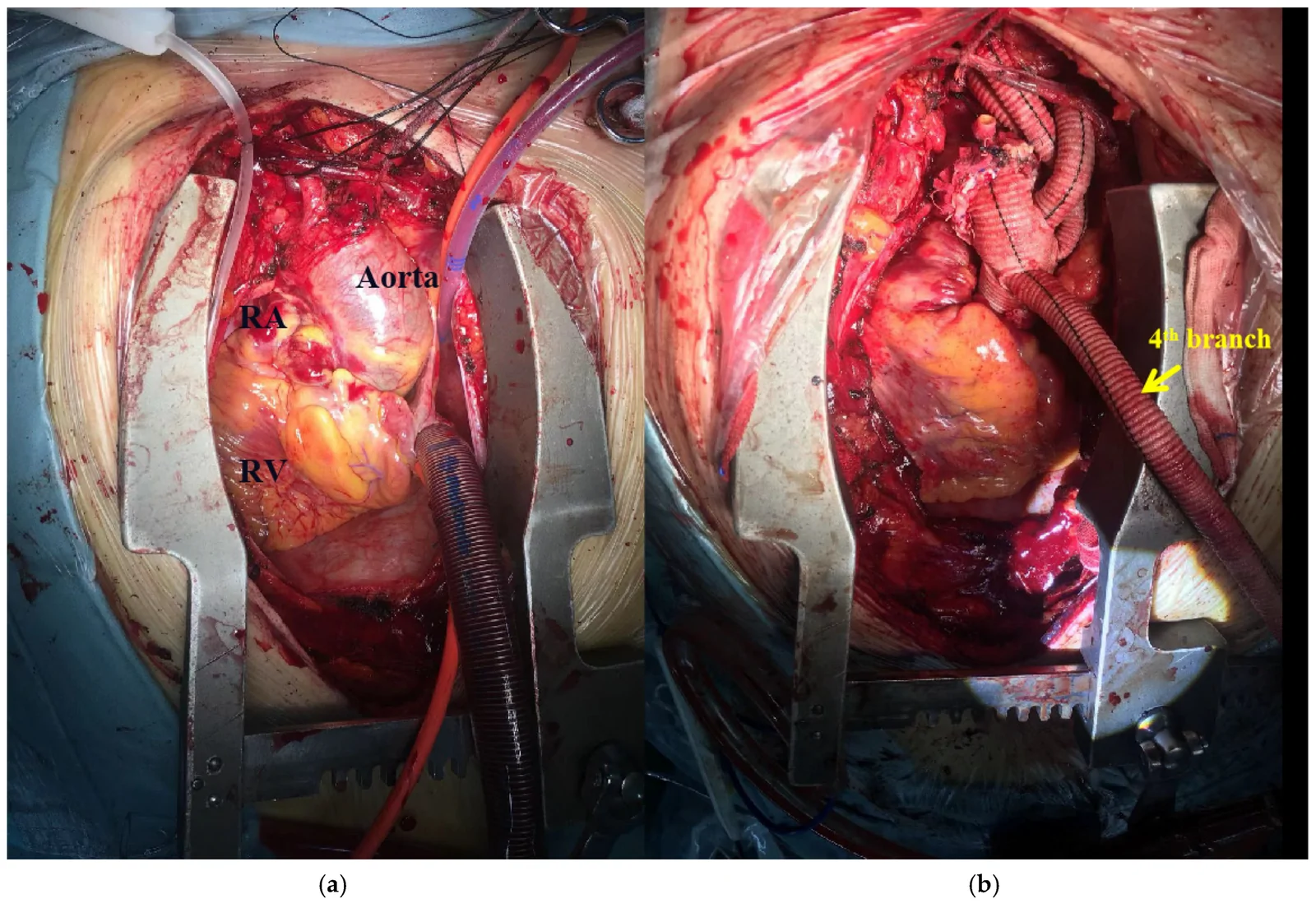
Intraoperative photographs of the surgical procedure. (**a**) Before aortic replacement; (**b**) after completion of the operation. Yellow arrow: the fourth branch of the aortic prosthetic graft. RA, right atrium; RV, right ventricle.

After median sternotomy, the left axillary artery was cannulated for arterial return and the right atrium, which lay on the left side of the midline in this mirrored anatomy, was cannulated for venous drainage to establish cardiopulmonary bypass (CPB). The ascending aorta was transected, and the non-coronary and right coronary sinuses were reconstructed with a prosthetic patch reinforced with autologous pericardium, with resuspension of the commissures using 5-0 polypropylene sutures. A 24 mm four-branched woven polyester graft (InterVascular, La Ciotat, France) with four side branches measuring 10, 8, 8 and 10 mm in diameter from the first to the fourth branch was then implanted in an inverted orientation (i.e., reversed relative to its intended use, with its proximal and distal ends swapped): its proximal end (originally the distal end) was anastomosed end-to-end to the aortic root with 4-0 polypropylene sutures reinforced with felt strips, and the fourth branch was connected to the arterial perfusion line. In the conventional orientation, the fourth branch would have been displaced posteriorly away from the operative field, rendering it unusable for perfusion and venting; implanting the graft in this inverted orientation-that is, with its own distal end anastomosed to the aortic root and its own proximal end used for the distal (arch) anastomosis-rotated the fourth branch to an anterior, accessible position (Figure 2). The right common carotid artery was then ligated, and the second branch of the graft was anastomosed to the right common carotid artery with 6-0 polypropylene. After cooling to a lowest nasopharyngeal temperature of 25.7 °C, unilateral antegrade selective cerebral perfusion (SCP) was administered through the second branch at a flow of 0.81 L/min/m^2^ for 23 min during lower-body circulatory arrest, with bilateral cerebral oximetry monitored throughout. During lower-body circulatory arrest, cerebral perfusion was maintained by unilateral antegrade SCP through the second branch-to-right-carotid anastomosis, while myocardial perfusion had already been restored: after completion of the proximal (root) anastomosis, the arterial perfusion line of the CPB circuit was connected to the fourth branch of the graft, and after thorough de-airing the distal end of the graft and the other three branches were clamped, thereby re-establishing antegrade coronary perfusion; the heart resumed beating, and no additional cardioplegia was required during the period of lower-body circulatory arrest. The right subclavian artery and the innominate artery were ligated, and the aortic arch was transected at the level of the innominate artery. A Cronus^®^ stent graft, 24 mm in diameter and 120 mm in length (MicroPort Endovascular, Shanghai, China), was deployed into the descending aorta, and the distal end of the prosthetic graft (originally the proximal end) was anastomosed to the stent graft in an end-to-end fashion. Full CPB flow was then restored and rewarming commenced. During rewarming, the third branch of the graft was anastomosed to the right subclavian artery and the first branch to the innominate artery (Figure 3). In summary, the four branches of the graft were assigned, from first to fourth, to the innominate artery, the right common carotid artery, the right subclavian artery, and perfusion/venting, respectively. Myocardial protection was achieved with 800 mL of 4:1 cold blood cardioplegia delivered immediately after aortic cross-clamping through directly visualized cannulation of the left and right coronary ostia, combined with topical cooling.

### 2.3. Postoperative Course and Follow-Up

The clinical timeline is summarised in Table 1. The patient regained consciousness and was successfully extubated within 24 h after surgery, with stable hemodynamics. The CPB, aortic cross-clamping, and SCP times were 247, 76, and 23 min, respectively, and the lowest nasopharyngeal temperature was 25.7 °C. The operation lasted 7 h and 12 min, with a total heparin dose of 240 mg, which was reversed with protamine at the end of CPB. Intraoperative blood loss was 300 mL, and 200 mL of 20% human serum albumin was infused intraoperatively. He was transferred out of the intensive care unit (ICU) on postoperative day 3 and discharged on postoperative day 23 (16 November 2023) with a satisfactory recovery.

**Figure 4:**
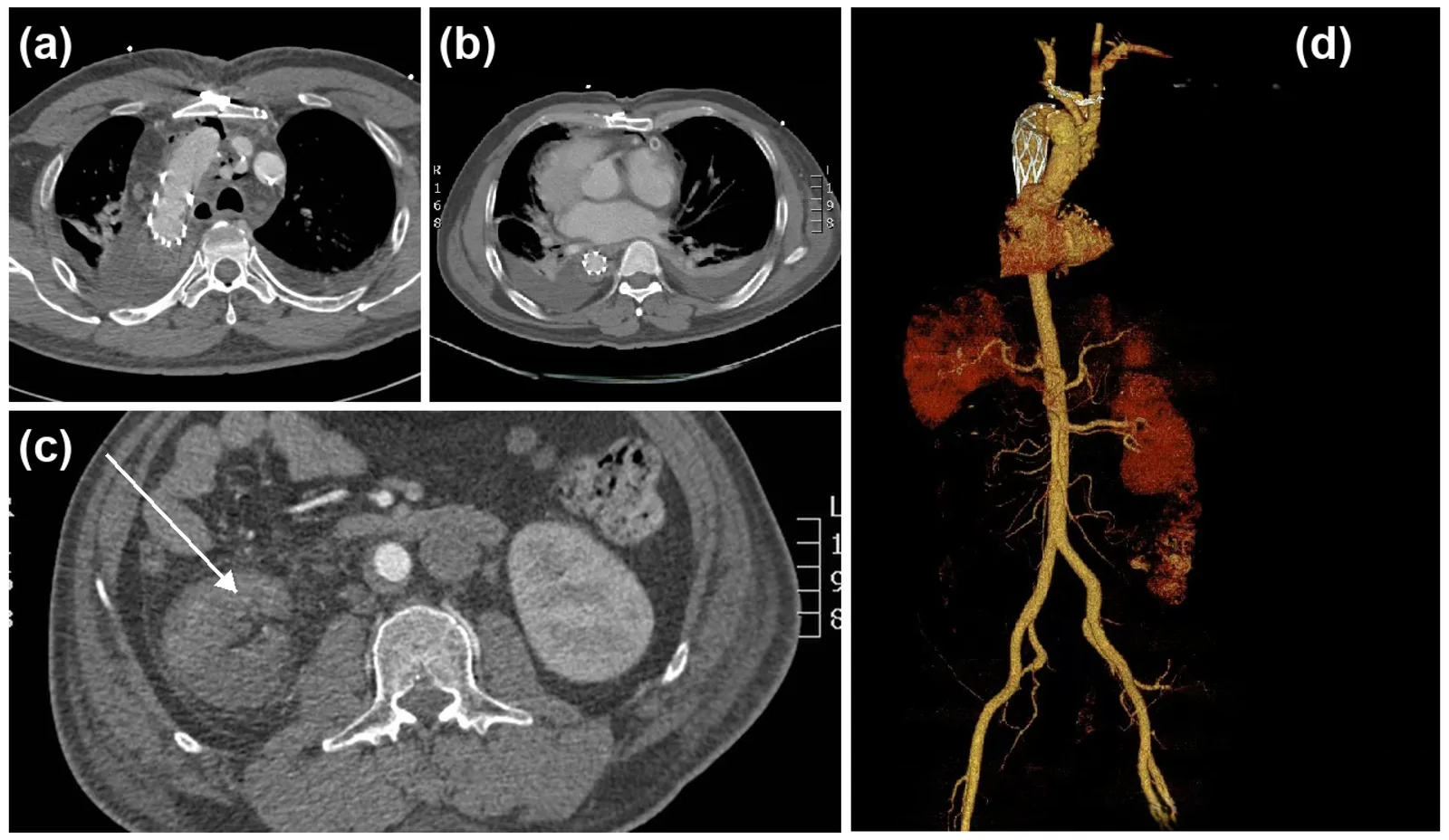
Postoperative imaging on day 3. (**a**) Axial CTA showing the prosthetic graft; (**b**) axial CTA showing the FET in position behind the cardiac silhouette; (**c**) axial CTA showing right renal malperfusion (arrow); (**d**) volume-rendered reconstruction showing the four-branched graft and the FET in situ.

Postoperatively, serum creatinine increased from 140 μmol/L before surgery to a peak of 297 μmol/L on postoperative day 1 (25 October 2023), corresponding to stage 2 acute kidney injury according to the KDIGO criteria (2.1 times the baseline value), and then decreased to 189 μmol/L at discharge; urine output remained adequate throughout the postoperative course, and no renal replacement therapy was required. On postoperative day 9, pleural effusion required thoracentesis, after which it gradually resolved; this accounted for the longer hospitalization. Postoperative echocardiography revealed the following findings: (1) changes consistent with ascending aorta replacement plus TAR with FET; (2) unobstructed blood flow in the aortic prosthetic graft and elephant trunk stent; and (3) mirror-image dextrocardia. Postoperative CTA on day 3 (Figure 4) showed: (1) changes consistent with ascending aorta replacement plus TAR with FET, without contrast extravasation; and (2) non-visualization of the right renal artery, accompanied by a significant reduction in right renal perfusion and surrounding exudative changes, consistent with right renal malperfusion.

**Figure 5:**
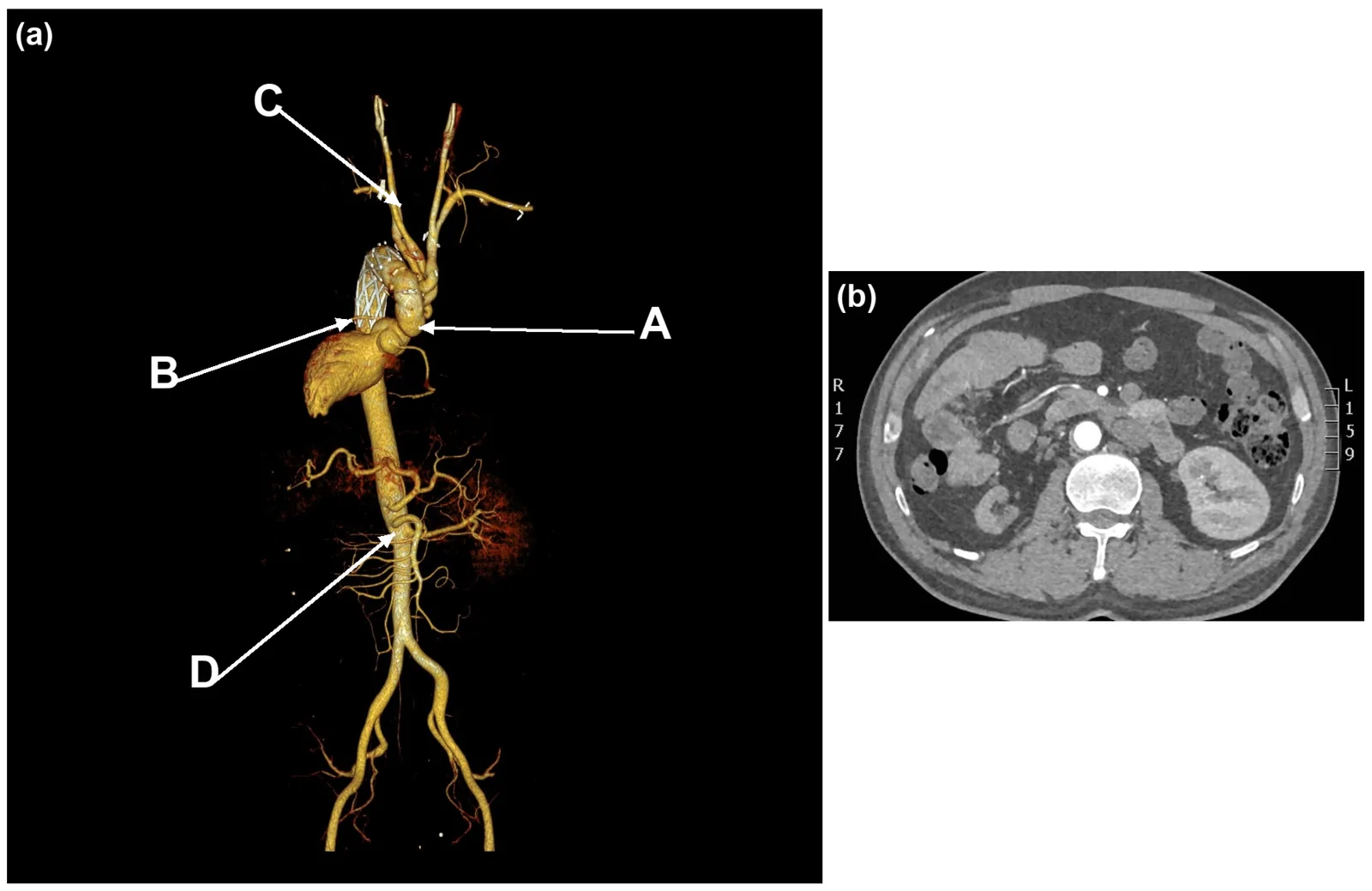
Follow-up CTA at 10 months (28 August 2024). (**a**) Volume-rendered reconstruction shows (A) the four-branched prosthetic graft, (B) the frozen elephant trunk stent graft in situ without endoleak, (C) the reconstructed supra-aortic branches, all patent, and (D) the descending aorta; (**b**) axial image showing the atrophic right kidney and the normally enhancing left kidney.

At the 10-month follow-up, CTA (28 August 2024) demonstrated that the four-branched graft and all three supra-aortic branch grafts were patent, that the FET was in position without endoleak, and that the peri-aortic thrombus had markedly regressed compared with the early postoperative scan; the right kidney was atrophic with a narrowed right renal artery, whereas the left kidney showed normal perfusion (Figure 5). At the telephone follow-up in October 2026, approximately 3 years after surgery, the patient was in good general condition, with stable renal function (serum creatinine approximately 130 μmol/L at local clinic visits), no readmission, and no aortic events or reinterventions; the next imaging follow-up has been scheduled.

## 3. Discussion

Acute type A aortic dissection in patients with SIT or mirror-image dextrocardia is exceedingly rare. To date, four cases have been reported: root and ascending aortic replacement in a 33-year-old man [12], ascending aortic replacement for dissection caused by blunt trauma in a 57-year-old man [13], the Bentall operation in a 37-year-old man [14], and ascending aortic replacement with tricuspid annuloplasty in a patient with severe tricuspid regurgitation [15]. In addition, TAR with FET was recently performed in a pregnant patient with SIT [10]. To the best of our knowledge, the present case is the first non-pregnant adult with SIT and mirror-image dextrocardia treated with ascending aorta replacement plus TAR with FET, and the first description of the inverted graft implantation technique for this scenario (Table 2).

The most challenging aspect of this operation was the anatomical mismatch between the commercially available four-branched aortic prosthetic graft and the patient’s unique anatomy, particularly the precise positioning of the fourth branch, which was required to maintain cerebral and cardiac perfusion throughout the procedure. With a conventional orientation, the fourth branch would be displaced posteriorly from its intended anterior position on the graft, rendering it non-utilizable. The inverted implantation technique adopted in this case provides a viable solution to this problem. A literature search of PubMed, Web of Science, and Embase up to October 2026, using the terms situs inversus, dextrocardia, aortic dissection, total arch replacement, and frozen elephant trunk, did not identify any other similar case, supporting the uniqueness of this technical approach.

In this patient, the aortic arch gave off three branches in a mirror-image arrangement; from proximal to distal, these were the innominate artery, the right common carotid artery, and the right subclavian artery, all of which were dissected and required reconstruction. The assignments of the four graft branches to the native vessels, together with the exact orientation of the graft before and after it was inverted, are illustrated in Figure 2. The brain-heart-first sequence, in which the second branch-to-right-carotid anastomosis was completed before circulatory arrest and used as the inflow for unilateral antegrade SCP, permitted cerebral perfusion during arch opening and the distal anastomosis, while the fourth branch served as the arterial inflow and venting line. Prior to surgery, it is imperative to obtain a comprehensive understanding of the patient’s medical condition and vital signs, the results of imaging and biochemical examinations, and a clear comprehension of the anatomical characteristics and variations present in patients with dextrocardia.

The primary challenges encountered during this operation were as follows: (1) to facilitate the surgical procedure, the surgeon positioned himself on the patient’s left side, deviating from the conventional surgical stance; (2) extracorporeal circulation was established through left axillary artery cannulation, rather than the typical right axillary artery approach; (3) meticulous dissection of the operative field was crucial to clarify the positional relationships among the superior and inferior vena cava, aorta, pulmonary artery, coronary ostia, atria, and ventricles, thereby reducing the risk of surgical complications; and (4) given the absence of a four-branched aortic prosthetic graft specifically designed for patients with mirror-image dextrocardia, we devised a strategy of inverting the proximal and distal ends of the graft, which effectively resolved the mismatch between the graft and the patient’s anatomical variation.

The decision to perform TAR with FET rather than isolated ascending or hemiarch replacement was based on the patient-specific anatomy. Although no intimal tear was identified in the ascending aorta or arch, the dissection involved all three arch branches, and the primary entry tear had been identified in the descending aorta on preoperative CTA. TAR allowed resection of the completely dissected arch and reliable reconstruction of all three supra-aortic branches, while the FET covered the distal entry tear and promoted distal aortic remodeling. Although extended arch replacement promotes favorable distal remodeling, whether it improves long-term survival compared with limited repair remains unresolved [8], and the extent of arch replacement should be individualized [6,7]. In this patient, with all three arch branches dissected and a presumed distal primary tear, we considered TAR with FET to be justified.

The choice of a two-component graft configuration also deserves comment. The combination of a four-branched woven polyester graft with a separate Cronus stent graft is the standard practice of the Sun’s procedure and has been widely adopted for TAR with FET in China; one-piece hybrid stent graft systems were not available at our institution. Beyond availability, this configuration proved particularly suitable for the mirrored anatomy: the diameter and covered length of the stent graft can be selected independently and matched to the descending aorta, the four branches can be assigned freely to the supra-aortic vessels in their mirrored positions, and the proximal anastomosis can be tailored to the reconstructed aortic root. Conversely, a one-piece hybrid stent graft could not have been inverted in the same way, because its stented component must be deployed in a fixed distal direction; in this mirror-image anatomy, its fourth (perfusion) branch would have remained posteriorly displaced and unusable. The only alternative would have been a custom-made, mirror-image-oriented one-piece prosthesis, and the emergency nature of acute type A aortic dissection leaves no time to wait for such customization. With the two-component configuration, only the prosthetic graft, and not the stented component, needed to be inverted: swapping the proximal and distal ends of the graft left the deployment of the stent graft unaffected.

With respect to cerebral protection, unilateral antegrade SCP was delivered through the anastomosis between the second branch of the graft and the right common carotid artery, with bilateral cerebral oximetry (left regional cerebral oxygen saturation 65–84%; right 76–91%) maintained within acceptable ranges, a lowest nasopharyngeal temperature of 25.7 °C, and an SCP duration of 23 min. In this mirrored anatomy, left axillary cannulation and the right common carotid artery substituted for the conventional right axillary route and left-sided arch branches, and the brain-heart-first sequence enabled re-establishment of cerebral perfusion before circulatory arrest [11].

The postoperative right renal malperfusion merits specific discussion. Early postoperative CTA showed non-visualization of the right renal artery with markedly reduced right renal perfusion and surrounding exudative changes, most likely reflecting dynamic obstruction or distal embolization related to the residual dissection, although a pre-existing renal arterial involvement could not be excluded. The patient was managed conservatively, and overall renal function recovered without renal replacement therapy. At 10 months, the right kidney had become atrophic with a narrowed right renal artery, whereas the left kidney maintained normal perfusion and compensated; overall renal function remained stable (serum creatinine approximately 130 μmol/L). This course underscores that renal malperfusion after surgery for acute dissection may initially be clinically silent and should be actively sought on early postoperative imaging, with close surveillance of renal function and renal artery status during follow-up.

Finally, extensive reconstruction of the supra-aortic branches with a four-branched graft carries a recognized risk of branch-graft complications, including thrombotic occlusion and anastomotic pseudoaneurysm [16]. In the present case, all three branch grafts were patent on both the early postoperative and the 10-month follow-up imaging, and no neurological symptoms were reported during follow-up.

This report has several limitations. First, it describes a single case, and the inverted graft technique was performed only once; its safety and reproducibility need to be verified in larger series. Second, the follow-up beyond 10 months was based on a telephone interview and local laboratory tests; the scheduled imaging follow-up will further clarify distal aortic remodeling and branch-graft status. Third, the present strategy is specific to the mirrored arch anatomy encountered in this patient and may not be directly generalizable to other laterality phenotypes.

## 4. Conclusions

Acute type A aortic dissection in a patient with SIT and mirror-image dextrocardia can be treated successfully with ascending aorta replacement plus TAR with FET. The success of this operation depended on accurately identifying the anatomical variations in the heart and great arteries, selecting the correct arterial cannulation site to establish extracorporeal circulation, and utilizing the inverted graft implantation technique to resolve the graft-anatomy mismatch.

## Figures and Tables

**Table 1:** Clinical timeline according to the CARE guidelines.

Time Point	Event
23 October 2023 (evening)	Sudden tearing chest and back pain; CTA confirmed acute type A aortic dissection, mirror-image dextrocardia, and situs inversus totalis; admission
24 October 2023	Emergency ascending aorta replacement plus TAR with FET using an inverted four-branched graft
25 October 2023 (day 1)	Peak serum creatinine 297 μmol/L (KDIGO stage 2 acute kidney injury), followed by a gradual decline; urine output adequate, no renal replacement therapy
27 October 2023 (day 3)	Step-down from ICU; CTA: graft and FET in position without contrast extravasation; right renal malperfusion identified
1 November 2023 (day 9)	Thoracentesis for pleural effusion; gradual resolution thereafter
16 November 2023 (day 23)	Discharge with satisfactory recovery; serum creatinine 189 μmol/L
28 August 2024 (10 months)	CTA: graft and all branch grafts patent; FET in position without endoleak; right kidney atrophic, left kidney normal
October 2026 (~3 years)	Telephone follow-up: good general condition; creatinine ~130 μmol/L; no readmission or reintervention; imaging follow-up scheduled

**Table 2:** Comparison of the present case with previously reported cases of acute type A aortic dissection in patients with situs inversus.

Study	Age/Sex	Anatomy	Etiology	Procedure	Cerebral Protection	Outcome
Niino et al., 2003 [12]	33/M	SIT + dextrocardia	Spontaneous	Aortic root and ascending replacement	Antegrade brain-isolated ECC (details not reported)	Uneventful
Magishi et al., 2006 [13]	57/M	SIT	Blunt trauma	Ascending replacement	Not reported (CPB with external cardiac massage)	Discharged 3 weeks after surgery
Kim et al., 2020 [14]	37/M	SIT	Spontaneous	Bentall operation	Not reported	Uneventful
Hachiro et al., 2022 [15]	Not reported	SIT	Spontaneous	Ascending replacement + tricuspid annuloplasty	Not reported	Uneventful
Yang et al., 2026 [10]	27/F (24 weeks of gestation)	SIT	Spontaneous	Ascending replacement + TAR + FET	Not reported	Fetal demise on day 1; mother well at 10-year follow-up
Present case	50/M	SIT + mirror-image dextrocardia	Spontaneous	Ascending replacement + TAR + FET (inverted four-branched graft)	Unilateral antegrade SCP via right common carotid artery, 23 min	Right renal malperfusion; overall renal function recovered, right kidney atrophic at 10 months; well at ~3-year follow-up

## Data Availability

Data will be made available on request.

## Supplementary Materials

The following supporting information can be downloaded at: https://www.mdpi.com/article/10.3390/jcm15218500/s1, The preoperative CTA cine loop (Video S1) is available online.

## Abbreviations

AAD	acute type A aortic dissection
CPB	cardiopulmonary bypass
CARE	CAse REport
CTA	computed tomographic angiography
FET	frozen elephant trunk
ICU	intensive care unit
SCP	selective cerebral perfusion
SIT	situs inversus totalis
TAR	total arch replacement

## References

[1] Maldjian P.D., Saric M. (2007). Approach to dextrocardia in adults: Review. AJR Am. J. Roentgenol..

[2] al-Khadra A.S. (1995). Images in cardiovascular medicine. Mirror-image dextrocardia with situs inversus. Circulation.

[3] Bohun C.M., Potts J.E., Casey B.M., Sandor G.G. (2007). A population-based study of cardiac malformations and outcomes associated with dextrocardia. Am. J. Cardiol..

[4] Czerny M., Grabenwöger M., Berger T., Aboyans V., Della Corte A., Chen E.P., Desai N.D., Dumfarth J., Elefteriades J.A., Etz C.D. (2024). EACTS/STS Guidelines for diagnosing and treating acute and chronic syndromes of the aortic organ. Ann. Thorac. Surg..

[5] Carbone A., Palladino R., Franzese M., Castaldo R., Ranieri B., Crisci G., Izzo R., Esposito G., Cittadini A., Schreurs B. (2024). Health-related quality of life in patients with aortic dissection: An unmet need. Curr. Probl. Cardiol..

[6] Carrel T., Sundt T.M., von Kodolitsch Y., Czerny M. (2023). Acute aortic dissection. Lancet.

[7] Czerny M., Schmidli J., Adler S., van den Berg J.C., Bertoglio L., Carrel T., Chiesa R., Clough R.E., Eberle B., Etz C. (2019). Editor’s Choice—Current options and recommendations for the treatment of thoracic aortic pathologies involving the aortic arch: An expert consensus document of the European Association for Cardio-Thoracic Surgery (EACTS) & the European Society for Vascular Surgery (ESVS). Eur. J. Vasc. Endovasc. Surg..

[8] Larsen M., Trimarchi S., Patel H.J., Di Eusanio M., Greason K.L., Peterson M.D., Fattori R., Hutchison S., Desai N.D., Korach A. (2017). Extended versus limited arch replacement in acute Type A aortic dissection. Eur. J. Cardiothorac. Surg..

[9] Zhu Y., Lingala B., Baiocchi M., Tao J.J., Arana V.T., Khoo J.W., Williams K.M., Traboulsi A.A.-R., Hammond H.C., Lee A.M. (2020). Type A aortic dissection—Experience over 5 decades: JACC historical breakthroughs in perspective. J. Am. Coll. Cardiol..

[10] Yang Y., Ge Y., Li C., Zhu J. (2026). Arch and frozen elephant trunk repair for aortic dissection in pregnancy with situs inversus totalis. Interdiscip. Cardiovasc. Thorac. Surg..

[11] Shen K., Tan L., Tang H., Zhou X., Xiao J., Xie D., Li J., Chen Y. (2022). Total arch replacement with frozen elephant trunk using a new brain-heart-first strategy for acute DeBakey type I aortic dissection can be performed under mild hypothermia (≥30 °C) with satisfactory outcomes. Front. Cardiovasc. Med..

[12] Niino T., Shiono M., Inoue T., Hata M., Sezai A., Negishi N. (2003). A case of acute aortic dissection type A in a patient with situs inversus. Ann. Thorac. Surg..

[13] Magishi K., Izumi Y., Ishikawa N., Kimura F. (2006). Stanford type A acute aortic dissection caused by blunt trauma in a patient with situs inversus. Ann. Thorac. Surg..

[14] Kim D.K., Lee J.M., Heo S.Y., Jung J.P., Park C.R., Lee Y.J., Lee S.C., Hwang S.K., Kim G.S. (2020). Acute type A aortic dissection in a patient with situs inversus totalis. Korean J. Thorac. Cardiovasc. Surg..

[15] Hachiro K., Kinoshita T., Shiraishi S., Suzuki T. (2022). Stanford type A aortic dissection with tricuspid regurgitation in a patient with situs inversus totalis. Kyobu Geka.

[16] Saltarocchi S., De Orchi P., Saade W., D’ABramo M., Chourda E., Romiti S., Vinciguerra M., Greco E., Miraldi F., Mazzesi G. (2022). Acute thrombotic occlusion of a brachiocephalic branch graft and pseudoaneurysm formation after debranching surgery for a non-A non-B aortic dissection. J. Card. Surg..

